# Elastography detected solid organ stiffness increased in patients with acromegaly

**DOI:** 10.1097/MD.0000000000014212

**Published:** 2019-01-18

**Authors:** Mehmet Bankir, Hilmi Erdem Sumbul, Ayse Selcan Koc, Derya Demirtas, Fettah Acibucu

**Affiliations:** a Department of Internal Medicine; b Department of Radiology, University of Health Sciences—Adana Health Practice and Research Center, Adana, Turkey.

**Keywords:** acromegaly, elastography, solid organ involvement, solid organ stiffness

## Abstract

Elastography is a method to examine the increase in solid organs stiffness (SOS), and there is no data in the literature regarding to its use in patients with acromegaly. In this study, we aimed to investigate the change of SOS in patients with acromegaly and to determine the parameters closely related to SOS in same patient groups.

We included 40 subjects with acromegaly and 40 healthy control subjects. In addition to routine renal, liver and thyroid ultrasonography (USG), SOS for 3 solid organs were measured by elastography. The participants of the study were divided into 3 groups as the control (Group-I), acromegaly patients with remission (Group-II), and acromegaly patients without remission (Group-III).

Insulin growth factor-1 (IGF-1) level significantly increased from Group-I to Group-III. Glucose, creatinine, albuminuria, alkaline phosphatase, TSH, and growth factor levels were highest in Group-III and statistically significance was found only between Group-I and Group-III. Liver, kidney and thyroid size and echogenicity were increased from Group-I to Group-III. Liver and renal stiffness and thyroid gland strain ratio significantly increased from Group-I to Group-III and these parameters were statistically different between all groups. In linear regression analysis, IGF-1 levels were independent determinants of SOS.

SOS values of acromegaly patients with active disease were significantly increased compared to both the control group and the acromegaly patients in remission phase. Serum IGF-1 levels were independently associated with SOS in these patients. SOS measurement should be part of a routine USG examination in patients with acromegaly, especially in patients during active disease.

## 1 Introduction

Acromegaly is a disease associated with growth hormone (GH) secreting pituitary adenoma, characterized by insulin-like growth factor-1 (IGF-1) increase, excess protein synthesis and excessive tissue growth due to these hormones.^[1,2]^ Chronically elevated levels of IGF-1 in patients with acromegaly cause structural and functional changes that are specific for the disease.^[1,3]^ Acromegaly is diagnosed by this typical appearance; acromegalic face, growth of hands and feet, acral enlargement, excessive increased IGF-1 level and advanced imaging method to diagnose pituitary adenoma.^[4]^ However, in these patients, growth of kidney, liver, pancreas, thyroid, and adrenal glands has been shown in recent studies to be associated with the effect of chronic IGF-1 increase on solid organs.^[2,5–9]^ However, recent ultrasonography (USG) methods other than volumetric growth in solid organs have not been evaluated in this patient group recently. Share wave elastography (SWE) and strain elastography (SE) are noninvasive, stable and cost-effective USG examinations used to assess tissue elasticity.^[10–12]^ It has been shown that the renal cortical stiffness (CS) determined by SWE is not affected by systemic and demographic parameters, and is associated with parenchymal disease and fibrosis.^[11–13]^ However, as far as we investigate, there is no data on the change in renal CS, liver stiffness (LS) or thyroid gland strain ratio (TG-SR) detected with SWE or SE in patients with acromegaly. We hypothesized that changes in cellular levels without solid organ involvement in patients with acromegaly could be manifested by solid organ stiffness (SOS) detected by SWE and SE.

Therefore, we aimed to investigate the changes of SOS obtained by SWE or SE examinations in patients with acromegaly in our study and to determine the parameters affecting SOS.

## 2 Methods

We included 40 patients with acromegaly (mean age: 42.3 ± 11.1 years, male/female: 27/13) and 20 healthy control subjects similar in age, sex and body surface area (mean age: 41.1 ± 9.4 years, male/female: 12/8) in our study. Current guidelines have been used in the diagnosis, treatment and classification of patients with acromegaly.^[4]^ The participants of the study was divided into 3 groups as the control or Group I (healthy controls), acromegaly patients with disease remission since 6 months or Group II (the patients with treated surgery and/or medical treatment and who had remission for acromegaly after these treatment), and acromegaly patients during active disease or group III (de novo patients or the patients with treated surgery and/or medical and who had not remission for acromegaly with these treatments). In Group III, 8 of the patients were de novo diagnosed and 12 patients did not enter the remission with surgical and/or medical treatment. Those with secondary or malignant hypertension (HT), calcific plaques, abdominal aneurysm or dissection, congestive heart failure, cerebrovascular disease, severe heart valve disease, inflammatory diseases, hematologic diseases, cancer, pregnancy, active thyroid disorder, renal and liver failure were also excluded from both groups. The Local Ethics Committee of Cukurova University approved the study protocol and each participant gave written informed consent.

After a detailed medical history and a complete physical examination, basic characteristics of patients such as age, gender, HT, diabetes mellitus (DM), current smoking status, coronary artery disease, family history, and hyperlipidemia, medical treatment, and body mass index (BMI) were recorded. White blood cell concentrations, hematocrit, and platelet count, fasting plasma glucose, blood urea nitrogen (BUN), creatinine, urinary albumin-creatinine ratio (ACR), aspartate aminotransferase (AST), alanine aminotransferase (ALT), alkaline phosphatase (ALT), gamma glutamine transferase (GGT), free T4, and thyroid stimulated hormones (TSH), were measured using an automated chemistry analyzer using commercial kits. Serum GH was assessed by an automated chemistry analyzer (Abbott Aeroset, MN) using appropriate commercial kits (Abbott) and reference value of GH was between 0.014and 5.219 ng/mL. Serum total IGF-1 level vas assessed by an automated chemistry analyzer (Abbott Aeroset, MN) using appropriate commercial kits (Abbott) and references value of IGF-1 vary according to age and sex. Serum GH and IGF-1 levels were measured the same time of USG examination for each acromegaly patient.

Liver, renal, and thyroid gland USG were examined with a high resolution Doppler ultrasound system (Philips EPIQ 7) equipped with a 5-1 and 12-5 MHz high resolution convex and linear converter (Philips Health Care, Bothell, WA), respectively. USG examinations were performed after a minimum 6 hours of fasting. All USG examination time was approximately 30–40 minutes. Subjects were evaluated by a 1 well-experienced radiology specialist for conventional, Doppler and SWE examinations. Specialist had more than 12 years of experience in USG studies and at least 500 SWE procedures in a year.

**Figure 1:**
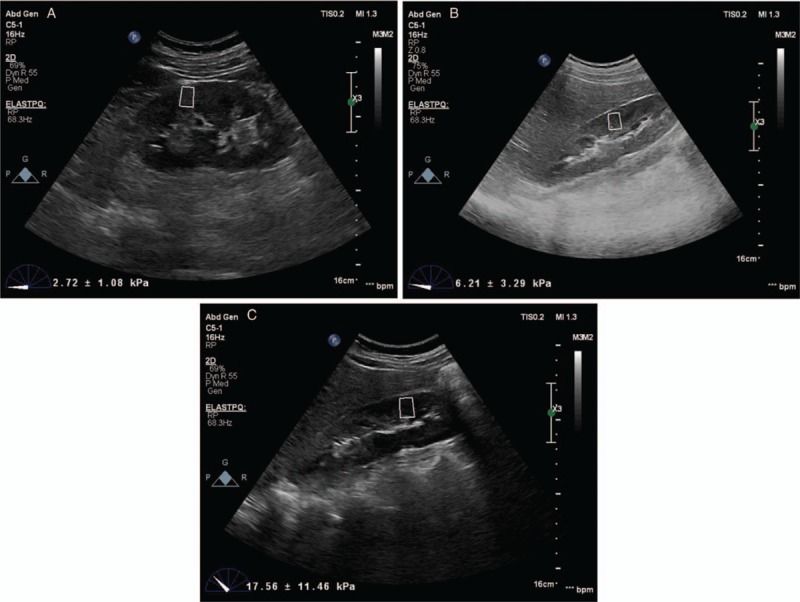
Renal cortical stiffness measurement by point shear wave elastography (A) normal renal cortical stiffness as 2.72 ± 1.08 kPa in control subject. (B) Increased renal cortical stiffness as 6.21 ± 3.29 kPa in patients with acromegaly with remission. (C) Significantly increased renal cortical stiffness as 17.56 ± 11.46 kPa in patients with acromegaly during active disease.

Kidney size, cortical thickness, and parenchyma echogenicity were assessed in gray scale. Kidney length was measured between upper and lower poles of the kidney. The distance between renal hilum and renal capsule was measured in the middle pole in coronal plane. Cortical thickness was measured between pyramid base of renal medulla in the middle and renal capsule. Shear wave elastography evaluation was obtained with 5-1 MHz convex abdomen probe by using elastography point quantification (ElastPQ) technique (Fig. 1A–C). Renal ultrasound was performed in the right and left lateral decubitus position. The least possible compression was applied to probe during ultrasound and probe was placed in a stable condition and patients were asked to hold their breath for a few seconds to minimize kidney movement with respiration. The measurements were taken after region of interest (ROI) was placed on targets on traditional renal ultrasound images. ROI was placed vertically on a renal cortex area without vessels or cysts. The main axis of ROI was adjusted to be parallel to the axis of renal pyramid (vertical to kidney surface). ROI target distance was maximum 8 cm and ROI constant box size was 1–0.5 cm. The applied compression was minimized during imaging to prevent mechanical pressure on kidneys. The contralateral kidney was imaged with the same technique. We obtained 6 valid measurements for each kidney and calculated the mean value. If reliability of measurement is low, the result will be seen with 0.00 kPa. Result is given as kPa value.

**Figure 2:**
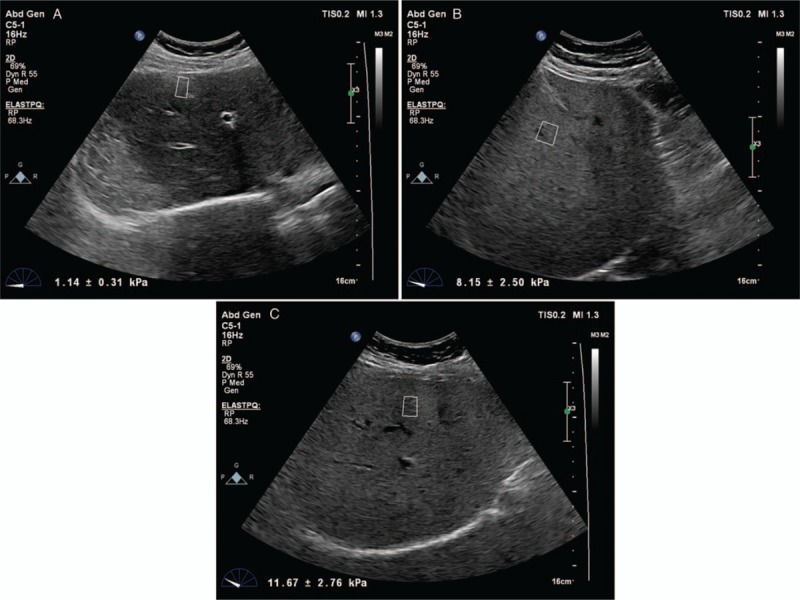
Liver stiffness measurement by point shear wave elastography. (A) Normal liver stiffness measurement as 1.14 ± 0.31 kPa in control subjects. (B) Increased liver stiffness measurement as 8.15 ± 2.50 kPa in patients with acromegaly with remission. (C) Significantly increased liver stiffness measurement as 11.67 ± 2.76 kPa in patients with acromegaly during active disease.

B-mode USG evaluation was first performed on the gray scale. Liver length and parenchyma echogenicity were assessed on gray scale. While the patient was in the supine position, the liver length was determined by the widest cranio-caudal measurement in the mid-clavicular area. Normally, the liver parenchyma has homogenous echo which is equal or slightly higher echogenic than normal renal cortex or spleen echogenity (grade 0). The presence of increased echogenicity in the liver parenchyma indicates mild fattiness (or grade 1) when the hepatic and portal venous walls are clearly visible but parenchyma being more echogenic compared with the kidney or spleen, moderate fattiness (or grade 2) when the hepatic and portal venous walls are undetectable and severe fattiness (or grade 3) when posterior attenuation, which means the posterior segments of the liver that cannot be assessed due to sonographically intense shadowing, and failure to detect diaphragm happens. LS was performed in the left lateral decubitus position using ElastPQ technique (Fig. 2A–C). During liver USG, the probe was compressed as lightly as possible and was placed in a stable position and the patient was asked not to breathe for a few seconds to minimize the movement of the liver with respiration. The measurement was calculated by placing the ROI on the target on the conventional USG image of the liver USG, after the target region was determined. The ROI was placed perpendicularly to a vascular-free or space occupying lesion-free zone. In our study, the ROI target distance was maximum 8 cm and the ROI fixed box size was 1–0.5 cm. In each case, 10 valid measurements were obtained from the varying segments of the liver parenchyma and the mean value was calculated. If the measurement reliability is low, a result of 0.00 kPa will be displayed. The result is expressed in kPa value.

**Figure 3:**
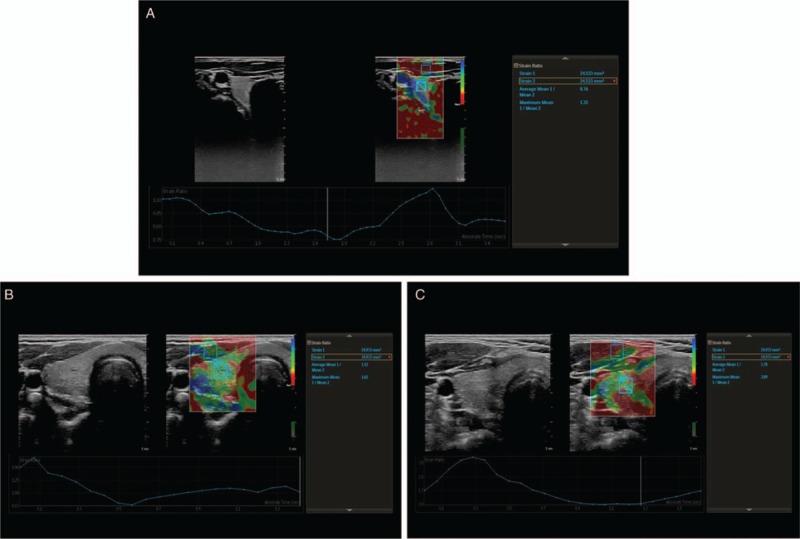
Thyroid gland strain ratio measurement by strain elastography. The first region of interest (ROI) (strain 1) was placed in the reference muscle tissue, and the other ROI was placed on the thyroid gland (strain 2). (A) The strain ratio was calculated as 0.76 and elastography color grades of the thyroid gland grade 3; blue (soft tissue) in control subjects. (B) The strain ratio was calculated as 1.12 and elastography color grades of the thyroid gland grade 2: green (intermediate tissue) in Acromegaly patient with remission. (C) The strain ratio was calculated as 1.78 and elastography color grades of the thyroid gland grade 1: red to yellow (hardest or hard tissue) in Acromegaly patient during active disease. ROI = region of interest.

Thyroid gland B-mode USG evaluation was performed while the patients were in the supine position. The thyroid gland thickness was calculated according to the maximum distance between the anterior and posterior walls, width was measured as the distance from the medial to the lateral wall in the transverse plane, and length was measured as the distance from the medial to the lateral wall in the longitudinal plane. The volume of each thyroid lobe was calculated with ellipsoid formula: length (cm) × width (cm) × thickness (cm) × 1/6 Л. The thyroid gland elastografi (TGE) evaluations were performed in the same position. Short repetitive manual compressions were applied to the thyroid gland with care to avoid anisotropy. The compression amount and uniformity were standardized using pressure graphics. After at least 3 compression-relaxation cycles, the SE calculations were performed based on the best images. The thyroid gland stiffness was graded automatically by our USG device. These grades were as follows: Grade 1: red to yellow (hardest or hard tissue); Grade 2: green (intermediate tissue); and Grade 3: blue (soft tissue) (Fig. 3A–C). The TG-SR calculation was performed on the same images. This calculation was compared with a reference muscle tissue that was taken from the strap muscles (sternohyoid or sternothyroid muscle) located at the anterior the thyroid gland. The first ROI (strain 1) was placed in the reference muscle tissue, and the other ROIs were placed on the thyroid gland (strain 2). The strain ratio (Strain 2/Strain 1) was automatically calculated (Fig. 3A–C). The TG-SR was compared with an adjacent reference tissue and semiquantitatively evaluated. Three consecutive measurements were made for each lobe, and mean values were calculated from the results from both lobes.

Statistical analysis: All analyses were made by using SPSS 22, 0 (Chicago, IL) statistical software. The distribution of continuous variables was evaluated and tested for being norm by Kolmogorov Smirnov test. Continuous variables in group data were referred as mean ± standard deviation. Categorical variables were referred as number and percentiles. Student t test or One-way ANOVA were used to compare continuous variables in groups. While Mann–Whitney *U* test or Kruskal–Wallis 1-way ANOVA test were used for not normally distributed samples. The chi-square (χ^2^) test was used to compare categorical variables. Pearson correlation analysis was used for single variable correlation analysis. Linear regression analysis was used to determine markers found in single variable analysis which were independent from parameters related to SOS. It was determined as statistically significant if *P* < 0.05.

## 3 Results

The participants of the study were divided into 3 groups as the control (Group I), acromegaly patients with remission (Group II), and acromegaly patients during active disease (Group III).

When clinical and demographic findings were compared among the study groups, all clinical and demographic findings were similar among the groups except for the presence of HT, DM and smoking, systolic blood pressure (SBP), diastolic blood pressure (DBP) and heart rate (Table 1). SBP and DBP values were found to be higher in Group II and III than Group I. Pulse was significantly higher Group III than Group I. Glucose, BUN, creatinine, urinary ACR, ALP, TSH, and GH levels were highest in Group III and statistically significance was found only between Group III and Group I (Table 1). Platelet count was the lowest in Group III and statistically significance was found only between Group III and Group I (Table 1). Serum IGF-1 level increased significantly from Group I to Group III. It was determined that serum IGF-1 levels were statistically different between all study groups (Table 1). Other laboratory findings were similar among the groups (Table 1).

**Table 1:**
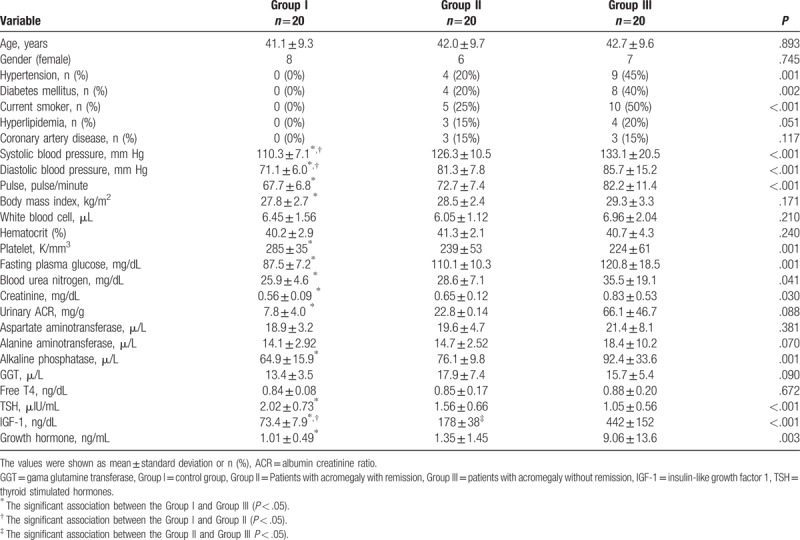
Demographic, clinical, and laboratory findings of the study groups.

**Figure 4:**
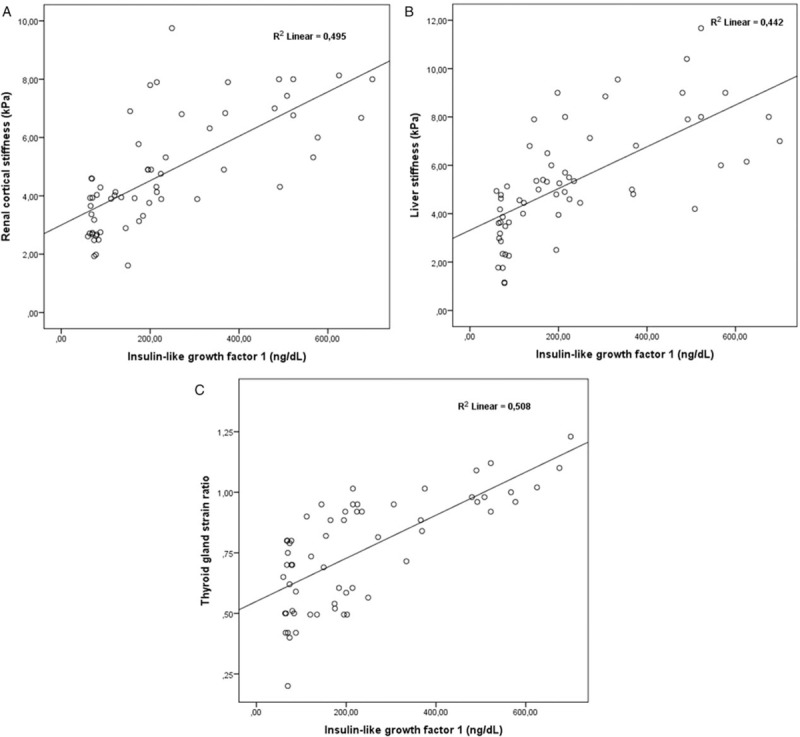
There is significant correlation between IGF-1 and (A) renal cortical stiffness, (B) liver stiffness and (C) thyroid stiffness or thyroid gland strain ratio.

When renal USG findings were compared between groups, kidney size, cortical thickness, renal echogenicity grade, and CS were highest in Group III and statistically significance was found only between Group I and Group III (Table 2). There was a positive correlation between CS and SBP, DBP, pulse, BMI, glucose, BUN, creatinine, urinary ACR, IGF-1, and GH levels. Platelet count was negatively correlated with CS. Levels of IGF-1 and SBP were independent determinants of renal CS in linear regression analysis (Table 3 and Fig. 4).

**Table 2:**
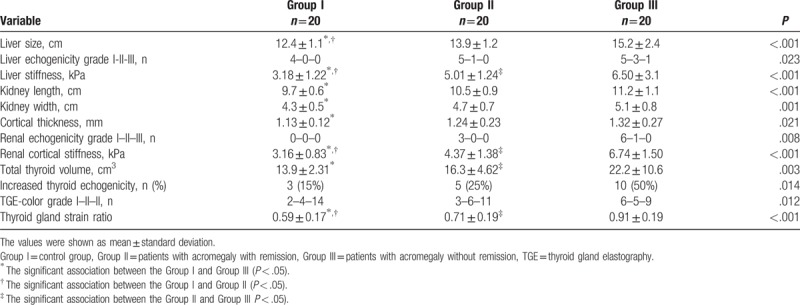
Liver, renal and thyroid ultrasound findings of study groups.

**Table 3:**
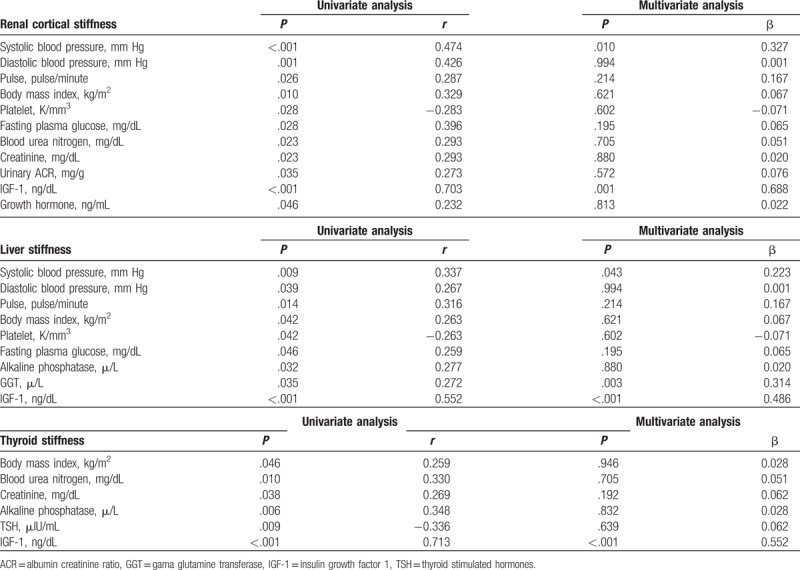
The parameters associated with renal cortical stiffness, liver stiffness and thyroid stiffness and linear regression analysis for parameters significantly correlated with these parameters.

When liver USG findings were compared between the groups; liver size, liver echogenicity grade, and LS values were significantly higher in Group II and Group III compared to Group I (Table 2). There was a positive correlation between LS and SBP, DBP, pulse, BMI, glucose, ALP, GGT, and IGF-1 levels. Platelet count was negatively correlated with LS. In linear regression analysis, SBP, GGT, and IGF-1 levels were independent determinants of LS (Table 3 and Fig. 4).

When thyroid USG findings were compared between groups; total thyroid volume, presence of increased thyroid echogenicity, TGE color grade and TG-SR values were significantly higher in Group III compared to Group I and Group II (Table 2). There was a positive correlation between TG-SR and BMI, creatinine, ALP, and IGF-1 levels. A level of TSH was negatively correlated with TG-SR. IGF-1 levels were independent determinants of TG-SR in linear regression analysis (Table 3 and Fig. 4).

## 4 Discussion

The main finding of this study is to demonstrate structural (conventional USG) and functional (with elastography techniques) changes in solid organs in patients with acromegaly. In our study, CS, LS, TGE color grade, and TG-SR values obtained for the first time with ElastPQ technique were found to be significantly higher in patients with acromegaly. SOS values are significantly higher in patients with acromegaly who are diagnosed de novo or having active disease despite treatment, than those who are in remission. In the acromegaly patients group, the increase of SOS by the elastography technique was shown for the first time. According to the results of our study, it was found that the mean LS, renal CS, and TG-SR values were 6.50 ± 3.1 kPa, 6.74 ± 1.50 kPa and 0.91 ± 0.19 in patients with acromegaly without remission. Furthermore, there was an independent relationship between IGF-1 levels and SOS values obtained by elastography.

SWE is a new USG technique developed for measuring tissue stiffness noninvasively and quantitatively. SWE is used for SOS assessment in many solid organs. These include liver, breasts, prostate, pancreas, testicles, thyroid, and kidneys.^[14]^ The CS and LS values obtained by the SWE examination are given as kPa, and the TG-SE obtained by SE is given as numbers.^[10,14–16]^ Recently, CS, LS, and TG-SR obtained from renal cortex, liver parenchyma, and thyroid gland by SWE and SE sequences can be used to examine parenchymal disease in these organs.^[10–14,17,18]^ However, due to the inability to perform the SWE and SE studies in some clinics, and the fact that the SOS limit value for healthy people is not clearly determined in studies, the data obtained by elastography is not stated in the USG report and measured only in some specific diseases and studies.

In our study, we assessed tissue elasticity with a new model USG device. We measured kidney and liver tissue stiffness with ElastPQ technique in liver and renal assessment. However, we did not use the same technique in thyroid gland because of the superficial localization of the gland, so that made the thyroid gland evaluation with the TG-SR and TGE color grade values, which gave a relative stiffness value using the SE method in the adjacent muscle.

Increased GH, IGF-1, increased renal hyperfiltration and excessive Na absorption in acromegaly patients lead to an increase in renal size and an increase in HT frequency.^[21]^ A study in acromegaly patients reported that kidney sizes and urinary ACR levels were higher in the active disease group than in the control group.^[6]^ In our study, both acute renal size and urinary ACR levels were higher in patients with acromegaly with active disease, similar to the previous study. As far as we investigate, there is no CS assessment obtained by SWE in patients with acromegaly in the literature. According to our results, renal CS obtained by SWE is increased in acromegaly with active disease. This relationship between renal CS and acromegaly points out that CS values should be evaluated in all patients with acromegaly, not only in patients who have nephropathy. This is important in clarifying the pathophysiology that changes in the renal parenchyma without renal involvement and without a decrease in eGFR in patients with acromegaly. For this reason, early collagen and renal fibrosis can be shown noninvasively with increased CS in patients with acromegaly and closer follow-up and more aggressive treatment can be done in this group of patients. In our study, it was thought that IGF-1 and SBP levels were most closely related to CS in patients with acromegaly, and that this parameter should be closely monitored for the development of renal parenchymal disease due to CS relationship.

The liver volume increase in patients with acromegaly was shown invasively by hepatic photo scan method in 11 patients with acromegaly in 1966.^[5]^ In the years that followed, USG studies showed liver volume increase,^[2]^ but recent USG methods were not used in this patient group. Liver enlargement has been shown to be slower after treatment with somatostatin analogs.^[2]^ In our study, similar to previous studies, it was shown that patients with acromegaly who had active disease had increased liver size and patients with remission were similar to the control group. However, in our study, no follow-up USG evaluation of patients was made and the effects of somatostatin analogs are unknown. In the literature, there is no data on the use of elastography in patients with acromegaly. In our study, in addition to previous literature data, there was an increase in LS value obtained by elastography examination in patients with acromegaly, which was closely related to IGF-1, SBP, and GGT levels.

Thyroid gland is clearly evaluated with USG in many diseases due to its superficial location. In our study, we performed TGE examination in addition to conventional thyroid USG. In patients with acromegaly, thyroid gland is affected frequently, which results in endocrine dysfunction, structural, and neoplastic changes.^[7]^ Total thyroid volume increases in patients with acromegaly but TSH level decreases.^[4,19,20]^ Treatment will normalize both volume and TSH levels.^[7,19,20]^ In addition, these changes are closely related to the IGF-1 level.^[20]^ In our study, TSH levels were decreased and thyroid volumes were higher in patients with active disease in accordance with previous studies. In patients with acromegaly, there is an increase in thyroid nodules in addition to an increase in thyroid volumes.^[4]^ Elastography examination is widely used for malign or benign differentiation of thyroid gland nodules.^[21]^

In patients with acromegaly, there are no studies that examine the thyroid gland stiffness only. We found that thyroid gland stiffness was significantly increased in patients with acromegaly in our study. Recently, Onal et al^[22]^ reported that the Achilles tendon thickness and tendon stiffness (grade II intermediate elasticity) obtained by SE were higher in patients with acromegaly with active disease. However, in the same study, it was reported that there was no relationship between serum GH and IGF-1 level and this increased stiffness in the Achilles tendon.^[22]^ In our study, Achilles tendon evaluation was not performed, but TGE evaluation was similarly performed and it was found that the tissue stiffness increased similarly to the previous study. Unlike the previous study, increased organ stiffness was independently associated with serum IGF-1 levels. The most important cause of this was related to the increase in collagen and protein synthesis on tissues caused by chronically high IGF-1 levels in patients with acromegaly.

## 5 Limitations

Our study has some important limitations. First of all, the number of patients is relatively small, and there is a need for studies with more patients. Our study was conducted in 2 patient groups with acromegaly, and patients with apparent nephropathy, liver, or thyroid disease were excluded. A more meaningful result could be obtained if an acromegaly group with nephropathy, active liver, or thyroid disease was added as a third group to the study. In our study, the tissue elasticity was measured noninvasively by SWE not to be unethical, these measurements were not compared with histopathological evaluation with renal, liver or thyroid biopsy. However, detection of changes at the cellular level and association with SOS with histopathological evaluation could be more meaningful. We cannot say that whether increased SOS values in acromegaly patients are due to fibrosis or to IGF-1 effects at cellular level. In our study, from other solid organs, breast, prostate, pancreas, and testicles were not evaluated and if these evaluations were made more meaningful findings could be obtained. There are many methods in the literature for evaluating renal and liver elastography. We performed this study with ElastPQ, a new high-resolution technique. This study may not be available at all centers. For this reason, it is necessary for researchers to know that our limit and average values are determined by this device.

## 6 Conclusions

Patients with acromegaly, SOS values are elevated without obvious evidence of involvement in solid organ functions such as liver and kidney. In patients treated for acromegaly and in patients with ongoing active disease, SOS values were significantly increased compared to both control group and patients with acromegaly with remission. This clearly shows that being in remission in these patients is very important. For this reason, SOS measurement should be part of the USG study in routine follow-up of patients with acromegaly if possible. Increased IGF-1 levels and increased SOS levels are closely related in patients with acromegaly, so that patients with higher IGF-1 levels need closer follow-up. In conclusion, SOS detected with ElastPQ technique can be used as a powerful, reliable, objective, noninvasive, reproducible, inexpensive USG study in the diagnosis and follow-up of solid organ involvement of acromegaly in clinical practice. However, we concluded that these results obtained in our study should be strengthened by new studies of different and more patients with acromegaly.

## Author contributions

**Conceptualization:** Mehmet Bankir, Hilmi Erdem Sumbul, Ayse Selcan Koc.

**Data curation:** Hilmi Erdem Sumbul.

**Formal analysis:** Mehmet Bankir, Hilmi Erdem Sumbul.

**Investigation:** Hilmi Erdem Sumbul.

**Methodology:** Mehmet Bankir, Hilmi Erdem Sumbul.

**Project administration:** Hilmi Erdem Sumbul.

**Resources:** Hilmi Erdem Sumbul.

**Software:** Mehmet Bankir, Hilmi Erdem Sumbul.

**Supervision:** Hilmi Erdem Sumbul, Derya Demirtas, Fettah Acibucu.

**Validation:** Hilmi Erdem Sumbul.

**Visualization:** Hilmi Erdem Sumbul.

**Writing – original draft:** Mehmet Bankir, Hilmi Erdem Sumbul, Ayse Selcan Koc.

**Writing – review & editing:** Hilmi Erdem Sumbul, Ayse Selcan Koc, Derya Demirtas, Fettah Acibucu.

Ayse Selcan Koc orcid: 0000-0003-1973-0719.
